# Liver Gene Transfer of Interkeukin-15 Constructs That Become Part of Circulating High Density Lipoproteins for Immunotherapy

**DOI:** 10.1371/journal.pone.0052370

**Published:** 2012-12-21

**Authors:** Maria C. Ochoa, Jessica Fioravanti, Erwin H. Duitman, Jose Medina-Echeverz, Asis Palazon, Ainhoa Arina, Juan Dubrot, Carlos Alfaro, Aizea Morales-Kastresana, Oihana Murillo, Sandra Hervas-Stubbs, Jesus Prieto, Pedro Berraondo, Ignacio Melero

**Affiliations:** 1 Center for Applied Medical Research, University of Navarra, Pamplona, Spain; 2 Department of Immunology and Cell Biology, Research Center Borstel, Borstel, Germany; 3 Department of Pathology, University of Chicago, Chicago, Illinois, United States of America; 4 Department of Otolaryngology, Stanford Cancer Center and Institute for Stem Cell Biology and Regenerative Medicine, Stanford University, Stanford, California, United States of America; McMaster University, Canada

## Abstract

Apolipoprotein A-I (Apo A-I) is a major component of high density lipoproteins (HDL) that transport cholesterol in circulation. We have constructed an expression plasmid encoding a chimeric molecule encompassing interleukin-15 (IL-15) and Apo A-I (pApo-hIL15) that was tested by hydrodynamic injections into mice and was co-administered with a plasmid encoding the sushi domain of IL-15Rα (pSushi) in order to enhance IL-15 trans-presentation and thereby bioactivity. The pharmacokinetics of the Apo A-I chimeric protein were much longer than non-stabilized IL-15 and its bioactivity was enhanced in combination with IL-15Rα Sushi. Importantly, the APO-IL-15 fusion protein was incorporated in part into circulating HDL. Liver gene transfer of these constructs increased NK and memory-phenotype CD8 lymphocyte numbers in peripheral blood, spleen and liver as a result of proliferation documented by CFSE dilution and BrdU incorporation. Moreover, the gene transfer procedure partly rescued the NK and memory T-cell deficiency observed in IL-15Rα^−/−^ mice. pApo-hIL15+ pSushi gene transfer to the liver showed a modest therapeutic activity against subcutaneously transplanted MC38 colon carcinoma tumors, that was more evident when tumors were set up as liver metastases. The improved pharmacokinetic profile and the strong biological activity of APO-IL-15 fusion protein holds promise for further development in combination with other immunotherapies.

## Introduction

There is much interest in the development of interleukin-15 for immunotherapy [1], [2]. This is because it inhibits activation induced T cell death [3], homeostaticaly increases lymphocyte numbers [4], [5], and up-regulates the function of NK cells [6], [7], [8], [9] and IKDC (interferon-producing killer dendritic cells) [10]. IL-15 is more a costimulatory molecule than a soluble cytokine in the sense that it is physiologically trans-presented [11] as a cell surface complex which is non-covalently attached with high affinity to IL-15Rα [8], [9], [11], [12].

The region of IL-15Rα involved in transpresentation of IL-15 has been identified as the sushi domain (so named because of structural resemblance with the roll shape of a popular Japanese dish) [13], [14], [15]. The binding of IL-15 to the sushi domain of IL-15Rα is believed to orient the molecule and improves the interaction with the IL-2Rβ/IL-2Rγ signaling receptors [13], [15]. Indeed, IL-15 coupled to the sushi domain has been engineered to increase bioactivity [15].

For cancer immunotherapy, IL-15 has created great expectations based on mouse data from injections of the soluble cytokine [1] or gene transfer [16], [17], [18]. However, the most striking effects of IL-15 are observed in combinatorial immunotherapeutic strategies such as those with adoptive T cell transfer [19] or vaccines [20], [21].

The pharmacokinetic profile of IL-15 as a soluble molecule is not favourable since such a small protein undergoes rapid renal clearance. To stabilize the molecule and provide trans-presentation, IL-15Rα-Fc chimeric proteins are conjugated *ex vivo* to IL-15. The resulting complexes are much more bioactive and exert more potent immunotherapeutic effects [22], [23], [24].

GMP-grade IL-15 has been tested in non-human primates mainly showing expanding effects on CD8 memory T cells and NK cells [25], [26]. Most effects are transient and cease following cytokine withdrawal [26] thus offering a promising overall safety profile. Accordingly, phase I trials have begun (NCT01021059).

In mice, the immunotherapeutic effects of IL-15 against tumors are dependent on NK and CD8 T cells [2], [9], [27]. Importantly, the effects of IL-15 are very different from those of IL-2, although both cytokines share similar receptors and attain similar effects on lymphocyte cultures [2]. For instance, IL-15 inhibits activation-induced cell death *in vivo*, whilst IL-2 induces the contrary [2]. Furthermore, the phenotypes of IL-15^−/−^ [28] and IL-15Rα^−/−^ mice [29] show that IL-15 is absolutely required for NK and NKT cell ontogeny as well as for homeostatic maintenance of CD8 memory T cells. On the contrary, IL-2^−/−^ mice show a totally unrelated phenotype with autoimmune inflammatory bowel disease [30].

Lipids are transported in plasma wrapped in apolipoproteins that coalesce forming lipoparticles. Apo A-I is a major constituent of high density lipoproteins (HDL), whose main function is to gather cholesterol from the tissues and take it back to the liver [31]. High plasma concentrations of HDL are linked to lower risk of cardiovascular events [31]. Apo A-I protein is an HDL constituent synthesized and secreted by hepatocytes and interacts with the surface scavenger receptor BI (SR-BI) broadly distributed in the organism [32].

Because of these features, Apo A-I has been used to stabilize interferon α to achieve sustained levels of the cytokine with a safer profile [33]. In these constructions, the cytokine is supposed to be facing the hydrophilic side, popping from the hydrophobic core of the lipoprotein.

In this study, IL-15 was fused to Apo A-I and gene-transferred to the liver by hydrodynamic injections as previously performed with several cytokine genes [34] including IL-15 [35]. We show that this chimeric gene encompassing IL-15 leads to more sustained serum concentrations than the concentrations achieved with non-fused IL-15. Furthermore, we co-transferred to the hepatocytes the sushi domain of IL-15Rα and the resulting complex showed enhanced bioactivity and as a monotherapy shows partial efficacy against colon carcinomas derived from the MC38 cell line.

## Materials and Methods

### Mice and Cell Lines

C57BL/6 and (6–10 weeks old) were purchased from Harlan Laboratories (Barcelona, Spain). IL-15Rα^−/−^ mice in C57BL/6 background [29] were kindly provided by Dr. Hans Schreiber (University of Chicago). IL-15Rα^−/−^ mice in background B6129S2 and their syngenic wild-type controls were purchased from Jackson Laboratories (Bar Harbor, Maine, US). MC38 is a colon adenocarcinoma cell line of C57BL/6 origin whose identity has been verified by Idexx Radil (Columbia, MO, USA. Case 6592-2012) and was provided to us by Dr. Karl E. Hellström (Seattle, WA) [36]. YAC-1 cells were obtained from American Type Culture Collection (Manassas, VA, USA) [35]. CTLL-2 is a stable subclone of cytotoxic T-lymphocytes originally isolated from a C57BL/6 mouse kindly provided to us by Dr. Juan J Lasarte (CIMA. Pamplona. Spain) [37].

### Ethics Statement

All animal procedures were conducted under Navarra Government recommendations and the protocol was approved by the Committee on the Ethics of Animal Research of the University of Navarra (study approval number 060/10). All surgery was performed under anesthesia, and all efforts were made to minimize suffering.

### Plasmids

The human interleukin-15 expression plasmid (pVkL**/**IL-15IRESneo), which carries a modified cDNA encoding for hIL-15 protein was produced and purified as described [35], [38] and will be called phIL15.

For sushi domain construction, complete mIL15Rα was cloned into the BamHI and NotI sites of the vector pcDNA™3.1 (Invitrogen, Carfsbed, CA) (pIL15Rα). The C-terminal part was removed by PCR (Uprim. TAA AGA GGG CCC TAT TCT ATA GTG and Lprim.GGG GTC TCT GAT GCA CTT GAG) and subsequent ligation of the product. The transgene in the expression plasmid was verified by sequencing. We will refer to this as pSushi. Apolipoprotein A-I plasmid (*apoa1*, GeneID:11806) was produced and purified as described [33] and will be called pApo.

### Apolipoprotein A-I and Interleukin 15 Gene Fusion Designs

PCR amplification was carried out using the sense primer FwATGmApoA1 (5′-ATGAAAGCTGTGGTGCTGGC-3′) on pApo as a template, and the antisense primer RvAscImApoA1 (5′-GGCGCGCCCTGGGCAG-TCAGAGTCTCGC-3′), introducing the restriction site for the AscI enzyme (GGCGCGCCC) in the 3′ end of apolipoprotein A-I gene, eliminating the stop codon and including a GAP peptide as linker of the two coded proteins. mApoA-I-AscI purified cDNA was cloned in the expression vector pcDNA™3.1/V5-His TOPO® TA (Invitrogen, Carfsbed, CA), which we will call pCMV-mApoA1-AscI.

Human interleukin 15 sequence was cloned in the expression vector pTrcHis2 TOPO® TA (Invitrogen, Carfsbed, CA) using 5′AATAATGGCGCGCCGAACTGGATAG-ATG-3′ (FwAscIhIL-15) and 3′ GCGGCCGCTCAGGACGTGTTGATGAAC -5′ (RvNotIhIL-15) primers that introduced a restriction site for AscI enzyme in 3′ and NotI in 5′. pTrcHis2-hIL-15 was digested with AscI and NotI and the AscI-hIL-15-NotI DNA fragment (345 nt) was obtained.

To carry out the gene fusion, plasmid pCMV-mApoA1-AscI was digested with the AscI/NotI enzymes (New England Biolabs). The ligation was performed with the open plasmid pCMV-mApoA1-AscI and the AscI-hIL15-NotI insert in a 1∶3 (vector:insert) ratio using T4 DNA ligase High Concentration and 2X Rapid Ligation Buffer (Promega, Wl). The resulting 6669-nucleotide plasmid will hereinafter be called pApo-hIL15. All plasmids were confirmed by sequencing the cloned genes.

For the fusion of mouse albumin with hIL15, the plasmid pApo-hIL15 was digested with AscI/XhoI and the insert containing the hIL15 was cloned into a the plasmid pALF [39] after removal of the sequence of IFN by AscI/XhoI digestion.

### Hydrodynamic Injections and ELISA

C57BL/6 or IL-15Rα^−/−^ mice received an intravenous injection (tail vein) of 10 µg of plasmid in a volume of 100 ml kg^−1^ using a 27-G needle at a rate of 0.4 ml s^−1^, as described [40]. The hIL-15 serum concentrations were assessed by ELISA (OptEIA BD Biosciences, San Jose, CA, USA). Concentration of mouse Apolipoprotein A-I were measured with an ELISA kit (Cusabio, Hubei, P. R. China).

### RT-PCR and Analysis of hIL15 and mSushi Expresion

Total RNA from mice livers was isolated from samples using TRI reagent (Sigma, Dorset, UK). The concentration and purity of samples were determined by absorbance at 260 and 280 nm with background correction at 320 nm in a spectrophotometer. RNA was treated with DNase I and retrotranscribed to cDNA with MMLV RT in the presence of RNase OUT (all the reagents Invitrogen, Carlsbad, CA, USA). The reaction was incubated for 1 hour at 37°C, denatured for 1 minute at 95°C and taken to 4°C. The samples were used immediately for PCR or stored at −20°C.

Murine actin was used to standardize gene expression. The mRNA values were represented by the formula 2Δ^Ct^, where ΔCt indicates the difference in the threshold cycle between mActin and the target genes (all reagents from BioRad, Hercules, CA, USA). Primers for quantitative real time RT-PCR for hIL15 were: (Fw hIL15) 5′-TGGGTGAATGTAATAAGTGATTTGAAA-3′ and (Rv hIL15) 5′-TTTCCTCCAG TTCCTCACATTCTT-3′, for actin: (FwmActin) 5′-(CGCGTCCACCCGCGAG-3′ and (RvmActin) 5′-CCTGGTGCCTAGGGCG-3′, and primers for quantitative real time RT-PCR for the sushi domain were: (Fw Sushi) 5′-AGACAGACACCCTGCTGCTC-3′ and (Rw Sushi) 5′- CCACGTTGGTGTTCTTGTTG-3′.

#### Isolation of HDLs by differential ultracentrifugation in sodium bromide gradient

24 hours after hydrodynamic delivery of pApo, pApo-hIL15 or pApo-hIL15+ pSushi plasmids, HDLs were isolated from plasma samples as described by Rodriguez-Sureda et al [41]. Electrophoresis and immunoblotting against Apo A-I were performed as described [33].

### IL-15 Bioactivity Assay

CTLL-2 cells were plated in a 96-well plate at 10^4^ cells/well and 1∶10 diluted serum obtained 24 h after hydrodynamic injections was added. Each serum sample was serially diluted 1∶2 twelve times. The plates were incubated for 2 days and subsequently the CTLL-2 cells were pulsed with 1 µCi of tritiated thymidine ([^3^H]TdR) 8 h before [^3^H]TdR incorporation was measured using a Topcount liquid scintillation counter (Packard).

### Electrophoresis and Apo A-I Immunoblotting

HDL^+^ fraction samples were separated in 4–20% Trishepes PAGE LongLife iGels (Nusep, Lane Cove, Australia) gradient gels, and transferred to nitrocellulose membrane (Whatman, Kent, UK). Apo A-I and hIL-15 were detected with Goat polyclonal anti-bodies (Santa Cruz Biotechnology, Santa Cruz, CA, USA) and a 1∶200 dilution Anti-goat IgG (whole molecule)- HRP conjugated (Sigma-Aldrich, St Louis, MO, USA) as secondary antibody [33].

### Isolation of Mononuclear Cells from Spleen and Livers

Spleens and livers were incubated in collagenase and DNase (Roche) for 15 min at 37°C, mechanically disrupted and passed through a 70 µm nylon mesh filter (BD Falcon, BD Biosciences). Dissociated cells from livers were centrifuged with Percoll^©^ (GE Healthcare, Chalfont St Giles, UK) at 35% (500 g, 10 min, 20°C) making a gradient in order to eliminate parenchymal cells. Erythrocytes were lysed with ACK buffer. For the study of CD8 T and NK cell proliferation, splenocytes were enriched in DX5**^+^** or CD8^+^ cells using immunomagnetic beads according to the manufacturer’s protocol (Miltenyi Biotec, Bergisch Gladbach, Germany).

### Antibodies and Flow Cytometry

Cells isolated from livers and spleens were pretreated with Fc-Block (anti-CD16/32, eBioscience, San Diego, CA, USA). Monoclonal antibodies to the following mouse antigens were conjugated to fluorescein isothiocyanate, phycoerythrin, phycoerythrin-cyanine7, allophycocyanin or biotin: CD3 (145-2C11), NK1.1 (PK-136), CD8 (53-6.7) and CD44 (IIM7) and BdrU (BD Biosciences, San Diego, CA, USA). Intracellular stainings were performed with Cytofix/Cytoperm^©^ buffer ((BD Biosciences, San Diego, CA, USA). Biotinylated antibodies were visualized with streptavidin-APC-Cy7. FACSCanto and FACSCalibur cytometers were used for cell acquisition, and data analysis was performed using FACS DiVa (BD Biosciences) and FlowJo 7.2.1 (Tree Star Inc., Ashland, OR, USA).

### CFSE Labeling of Cells, Adoptive Transfer and BrdU Assessment of Proliferation *in vivo*

Enriched DX5^+^ or CD8^+^ cells were washed twice with phosphate-buffered saline and resuspended at 10^7^ cells ml^−1^ in 5 µM CFSE (Sigma-Aldrich) for 15 min at room temperature. 3×10^5^–5×10^5^ stained cells were transferred to mice by the retro orbital sinus. The proliferation of adoptively transferred NK and CD8 T lymphocytes was analyzed 72 h after the transfer by CFSE dilution using a FACSCalibur cytometer. For BrdU incorporation *in vivo* and detection by flow cytometry, we used the FITC BrdU flow kit (BD Biosciences) according to manufacturer instructions. Intraperitoneal injections of BrdU were provided three times every 24 h with doses of 1.5 mg.

### *In vivo* Tumor Growth

For the subcutaneous models, C57Bl/6 mice received a s.c. injection of MC38 cells (5×10^5^ per mouse). On the indicated days, mice were treated with a hydrodynamic injection with the plasmids. Tumor diameters were measured using an electronic caliper every 2 to 4 days, and tumor size was determined by multiplying perpendicular diameters.

For the intrasplenic tumor model, C57BL/6 mice were anesthetized and a small incision in peritoneum was performed to access the spleen. MC38 cells were injected in the spleen (5×10^5^ per mouse) in 50 µL of PBS. On day 1, mice were treated with the corresponding plasmids by hydrodynamic injection. Mice were observed daily and were sacrificed when one of the mice presented impaired mobility. Following sacrifice, tumor nodules in spleen and liver metastases were counted and measured. Mice were weighed as well as various excised organs upon necropsy.

## Results

### Fusion Proteins Encompassing IL-15 and Apo A-I are more Stable in Plasma and Partly become Complexed with HDL

IL-15 renal clearance is rapid due to the small molecular size of the protein. In order to stabilize IL-15 in circulation, constructions that encompassed Apo A-I lipoprotein and human IL-15 were prepared. Figure S1 represents the expression plasmids in which the indicated genes are placed under the transcriptional control of the CMV promoter. The sushi domain of IL-15Rα was also cloned in a similar expression cassette.

C57BL/6 mice were injected by hydrodynamic infusions with the expression plasmids as single agents or in combinations to achieve expression of the encoded proteins in the liver [35]. Since Apo A-I is naturally biosynthesized in the liver, local production of the secretable transgene products was expected to reach circulation. IL-15Rα sushi domain has been reported to conjugate with IL-15 non-covalently during protein processing [13], [42].

**Figure 1:**
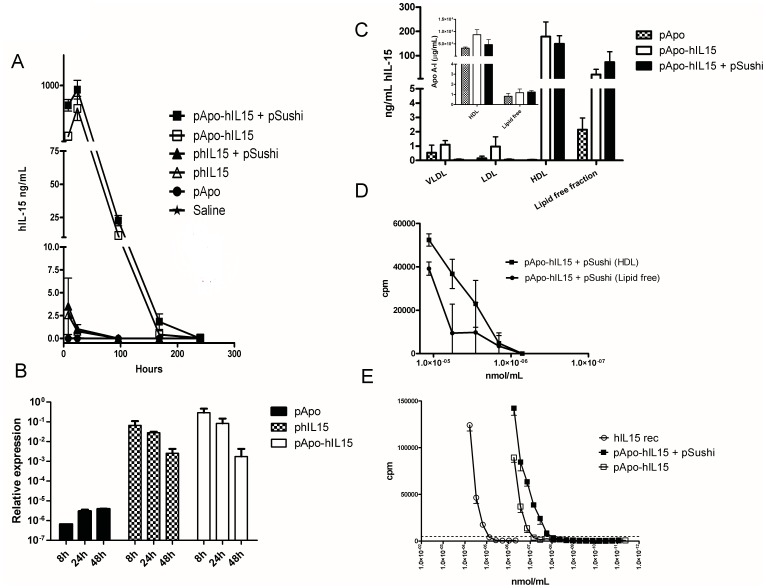
Liver gene transfer of APO-IL-15 and IL-15Rα sushi domain results in extended half-life and enhanced bioactivity. (A) Mice (n = 3 per group) were hydrodynamically injected with the indicated expression plasmids and serum concentrations of human IL-15 were sequentially monitored by ELISA. Mean ± SEM from one representative experiment out of three similarly performed. (B) IL-15 and Apo A-I mRNA expression quantified by real-time RT-PCR referred to β actin at the given time-points following hydrodynamic injection of the indicated plasmids as in A. (C) Plasma samples of mice hydrodynamically treated with the indicated plasmids were fractioned by centrifugation into the different lipoprotein particles and lipid-depleted plasma. IL-15 content in each fraction was monitored by ELISA. Results are from two pooled experiments performed with 4 and 6 mice per group. The inset represents the Apo A-I concentration in the indicated fractions. (D) IL-15 bioactivity measured on CTLL2 cells by ^3^H-Thy incorporation when testing the indicated dilutions of the serum and the HDL concentrated fractions from equivalent volumes of serum. Concentration were adjusted to nmol/mL using IL-15 concentration as assessed by ELISA. (E) CTLL-2 proliferation in response to sequentially diluted plasma samples in which IL-15 content had been measured by ELISA as in D. When indicated, mice had been hydrodynamically given pApo-hIL15 with or without combination with a plasmid encoding the IL-15Rα sushi domain. Data represent mean±SEM of four-replicated samples. The experiment was repeated at least three times with comparable results. Commercial human rIL-15 is included as a positive control and the discontinuous line represents thymidine incorporation to CTLL2 exposed to the serum of a mouse treated with pApo as a negative control.

Figure 1A shows the time courses of the serum concentrations of human IL-15 following hydrodynamic injections of the plasmids. As can be seen, conjugation to Apo A-I dramatically extends the pharmacokinetic profile and reaches much higher concentrations of the transgene product than non-fused IL-15. Liver mRNA from the expression plasmids was quantified by RT-PCR and found to correlate with peak protein expression (figure 1B). In addition, pSushi RNA was also detected by RT-PCR in similar samples, when pSushi was co-administered (figure S2).

Differential centrifugation of the plasma of the mice to separate the lipid fractions indicated that immunoreactive IL-15 by ELISA was observed both in high density lipoproteins (HDL) and in lipid-free plasma upon liver gene co-transfer of APO-IL-15 and IL-15Rα sushi domain, or upon a single hydrodynamic injection of the APO-IL-15-encoding plasmid (figure 1C). This indicates that the chimeric protein circulates in serum both free and complexed to HDL. The absence of HDL in the lipid free plasma fractions was checked by ELISA quantification of Apo A-I (inset to figure 1C).

These data were confirmed using HDL fraction and lipid-free plasma to test IL-15 bioactivity on CTLL2 cells. As can be seen in figure 1D both fractions contained bioactivity, but a comparison on nmol/mL basis indicates that the HDL complexed fraction is slightly more bioactive.

### Gene Co-transfer of the Sushi Domain of IL-15Rα Increases APO-IL-15 Bioactivity and thereby Increases Numbers of NK and CD8 T Cells in the Spleen and in the Liver

Bioactivity of the IL-15 molecules was characterized on a molar basis in functional tests on the proliferation of CTLL-2 cells (figure 1E). A one log difference was observed when we compared the IL-15 bioactivity from serum samples drawn from mice transferred with APO-IL-15 as a simple plasmid or co-transferred with the sushi domain of IL-15Rα. This finding is in line with the literature showing the enhanced effect of IL-15 in combination with the sushi domain [2], [10], [18], [42]. Of note, Apo A-I or other HDLs components do not hinder the IL-15/Sushi domain interaction, allowing the enhancement of bioactivity.

Given the fact that IL-15 is detected both in the HDL and the lipid free plasma fractions (figure 1C and D), we performed experiments to see if IL-15 were released from HDL upon overnight incubation at 37°C in PBS. We found that almost none of the HDL-complexed IL-15 was released into the buffer (data not shown).

**Figure 2:**
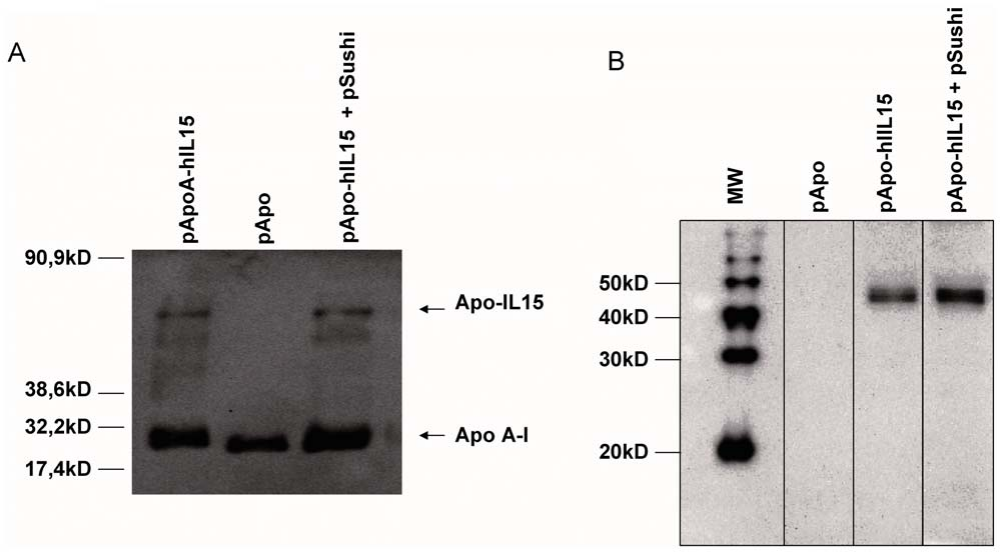
Western blot analysis of the circulating APO-IL-15 fusion protein. (A) Plasma HDL fractions from figure 1C were immunoblotted with an anti-Apo A-I polyclonal Ab. A shift in molecular weight indicated the chimeric proteins and the electrophoretic migration molecular weight standards are indicated. (B) Samples as in A were immunoblotted with an anti-IL15 polyclonal antibody and lanes with prestained molecular weight markers were run in parallel, indicating a fusion protein with a relative molecular weight between 40 and 50 kD.

In figure 2A, an immunoblot of the HDL fractions of the mice was probed with an anti-Apo A-I polyclonal Ab that showed a shift in molecular weight from the abundant non-complexed Apo A-I when the APO-IL-15 sequences had been transferred by hydrodynamic injection. This finding further indicates that APO-IL-15 was incorporated in part into circulating HDL. Furthermore, we developed similar blots with an anti-IL15 antibody that also showed a compatible molecular weight for the fusion form of IL-15 in the corresponding HDL fractions from mice in which pApo-hIL15 plasmids had been hydrodynamically injected (figure 2B).

In summary, IL-15 in covalent conjugation with Apo A-I complexes with HDL and extends its half-life. In addition, gene transfer of the sushi domain of IL-15Rα enhances the bioactivity of the cytokine.

One of the intended functions of Apo A-I is to increase molecular weight (figure 2A and B) is such a way that it surpasses the renal filtration threshold and hence extends half-life. This can be achieved to a similar extent with other fusion patners, such as albumin (figure S3), even though the biodistribution of the lipid-complexed IL-15 could be different. Another important question was if HDL from mice receiving plasmids encoding Apo-hIL15 preserved their anti-inflammatory and cholesterol transporting functions. As can be seen in figure S4, such HDL fractions preserved their ability to counteract TNFα induction of VCAM on mouse endothelial cells and promote the efflux of cholesterol from 3T3 cells.

**Figure 3:**
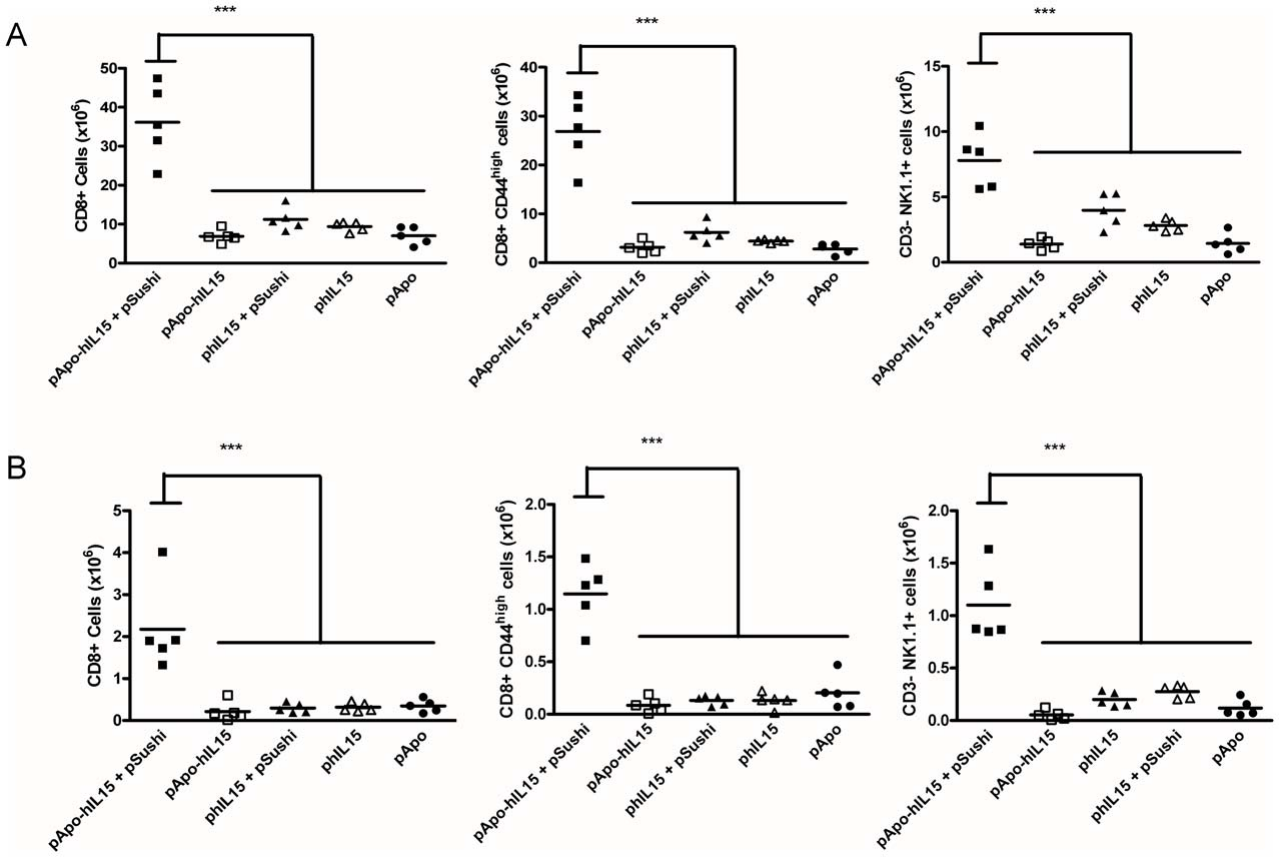
Figure 3. Gene transfer of APO-IL-15 and IL-15Rα sushi domain increase memory-phenotype CD8^+^ T cells and NK cells. (A) Absolute numbers of the indicated lymphocyte subsets in the spleen of mice treated with the indicated plasmids 5 days before splenectomy. Each point shows an individual mouse. This experiment was repeated twice with comparable results. (*** indicates p<0.0001 in a one-way ANOVA test followed by Bonferroni corrections). Figure S5 shows a time course analysis of these parameters (B) Experiments as in A measuring numbers of intrahepatic mononuclear leukocytes with the indicated surface phenotypes. Each point represents an individual mouse (*** indicates p<0.0001 in a one-way ANOVA test followed by Bonferroni corrections).

Importantly, mice which received pApo-hIL15+ pSushi showed a marked increase of CD8β^+^ T cells, CD44^high^ CD8^+^ memory T cells and NK cells in the spleen if analyzed five days following gene transfer. This finding as shown in figure 3A indicates that co-transfer with the sushi domain increases *in vivo* bioactivity. A time course (figure S5A) of an independent series of experiments indicates that the peak of the effects on immune system cells takes place around four days after the hydrodynamic injection of the plasmids. As previously reported [42], the IL-15Rα sushi domain also enhances the activity of IL-15 without Apo A-I. Of note, pSushi transference lacked by itself any kind of detectable effects (data not shown).

Liver lymphocytes followed a similar trend with a dramatic accumulation of memory CD8 T lymphocytes and NK cells, as shown in figure 3B. Indeed, the time course was similar with a peak around day 4 after plasmid injection (figure S5B). Sequential follow-up of peripheral blood mononuclear lymphocytes in treated groups of mice showed also these changes (figure S5C).

When testing the natural killer cytotoxic activity of spleen cells harvested three days following treatment with the pApo-hIL15+ pSushi in comparison to that of mice treated with pApo A-I, a clear increase in activity against YAC-1 target cells was observed (figure S6).

### Gene Co-transfer of APO-IL-15 and IL-15Rα Sushi Domain Induces the Proliferation of CD8^+^ and NK Lymphocytes

**Figure 4:**
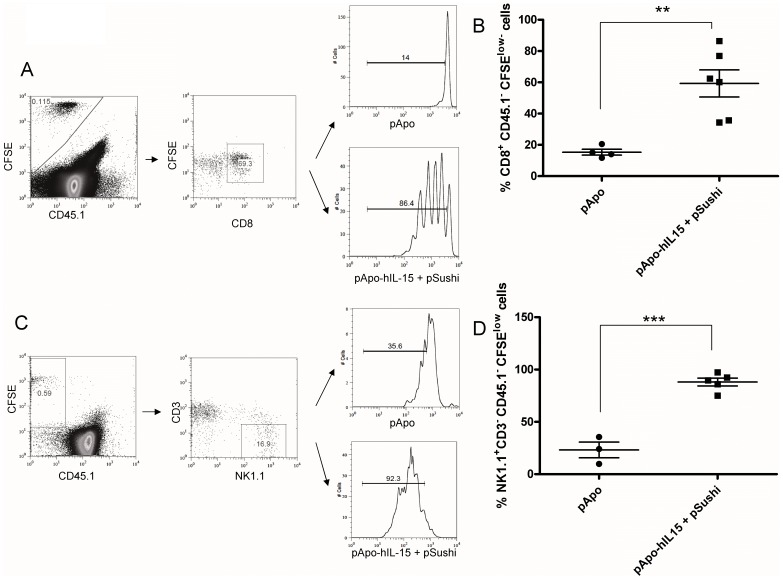
APO-IL-15 and IL-15Rα sushi domain gene co-transfer induces proliferation of adoptively transferred CD8 and NK lymphocytes. Mice were gene-transferred by hydrodynamic injections of the indicated plasmids which were 24 h later intravenously injected with CD8^+^ T cells or NK1.1^+^ NK cells preloaded with CFSE. Proliferation was monitored by CFSE dilution of the indicated electronically gated lymphocyte subsets (dot plots A and C). Results pooled from two independent experiments included two mice per experimental group (B and D). Figure S7 shows proliferative effects on endogenous lymphocytes studied by BrdU intake.

Accumulation of CD8 memory T cells and NK cells could be the result of enhanced proliferation. To study this possibility, CFSE-labeled NK and CD8 T cells were infused into mice one day following hydrodynamic injection of control plasmids or the pApo-hIL-15+ pSushi combination. As is shown in figure 4, most adoptively transferred cells undergo at least one cycle of proliferation in response to gene transfer of pApo-hIL15+ pSushi. Therefore, proliferation contributes at least in part to accumulation of such lymphocytes. To evaluate the induction proliferation on endogenous CD8^+^ T cells and NK cells, we performed experiments in which the mice were treated with BrdU and splenocytes stained for incorporation of this compound into the DNA of each gated lymphocyte subset. As it can be seen in figure S7, there was a clear stimulation of a proliferative response in both endogenous lymphocyte subpopulations upon treatment with pApo-hIL15+ pSushi.

### Gene Co-transfer of APO-IL-15 and IL-15Rα Sushi Domain Partially Rescues the NK and CD8 Phenotype of IL-15Rα^−/−^ Mice

**Figure 5:**
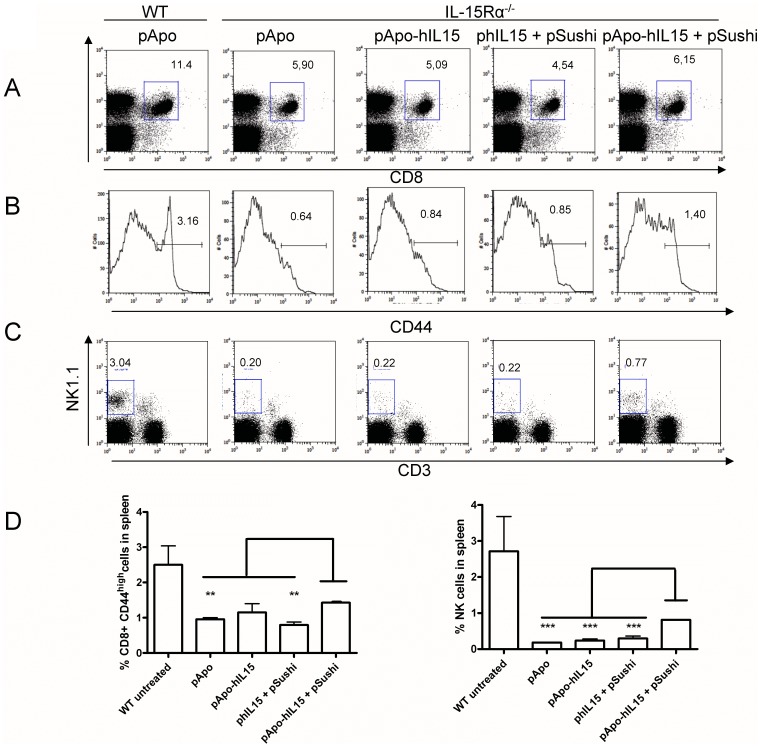
Partial rescue of the phenotype of IL-15Rα^−/−^ mice in NK cells and memory-phenotype CD8 cells. WT C57Bl/6 mice (WT) or IL-15Rα^−/−^ syngenic mice (IL-15Rα^−/−^) were injected with the indicated plasmids by hydrodynamic injection. Four days later splenocytes were immunostained. (A) CD3^+^ CD8^+^ dot plots with the percentage of events in the gated rectangles. Results reflect the percentage referred to total spenocytes (B) Histograms showing CD44 specific immunofluorescence on the cells gated in A to assess the percentage of CD44^high^ CD8^+^ memory-phenotype T cells. (C) CD3 NK1.1 double staining showing the percentage of events in each dot plot quadrant. Histograms and dot plots were taken from a representative individual mouse. (D) Grouped sets of data from other individuals. Confirmatory results in a series of mice in B6129 background are presented in figure S8.

IL-15^−/−^ and IL-15Rα^−/−^ mice are virtually devoid of NK and NKT cells and experience a drastic reduction in CD8 memory T cells [28], [29]. We intended to see if pApo-hIL-15+ pSushi liver gene transfer could modify the phenotype of IL-15Rα^−/−^ mice. Figure 5 shows the normal proportions of NK and CD8 cells in the liver of WT C57Bl/6 mice. IL-15Rα^−/−^ mice, even if transduced with pApo control plasmid four days before, showed negligible levels of NK (CD3^−^ NK1.1^+^) and NKT (CD3^+^ NK1.1^+^) cells. Transfer of phIL-15 and pApo-hIL15 only marginally increased these numbers. However, the combination of pApo-hIL15+ pSushi enhanced the number of NK cells, but not as much as NKT cells. These results were reproducible in the spleen of three individual mice per group as shown in figure 5B. Moreover, we have extended these results to IL-15Rα−/− mice in B6129S2 background using five mice per group as shown in figure S8A and B. In this background, the recovery of CD8 T cells with a memory phenotype was remarkable and NK cells, at least partially recovered in most cases, were identified as NKP46^+^DX5^+^CD3^−^.

We performed these experiments also with a plasmid encoding a full length pIL-15Rα instead of only the sushi domain. As can be seen in figure S8C, pSushi was superior to pIL-15Rα indicating that a sequence upstream the sushi domain somehow interferes with trans-presentation as suggested by previous reports [14], [42].

### Therapeutic Effects of Liver Gene Co-transfer with APO-IL-15 and IL-15Rα Sushi Domain on Subcutaneously and Intrasplenically Injected MC38 Colon Cancer Cells

**Figure 6:**
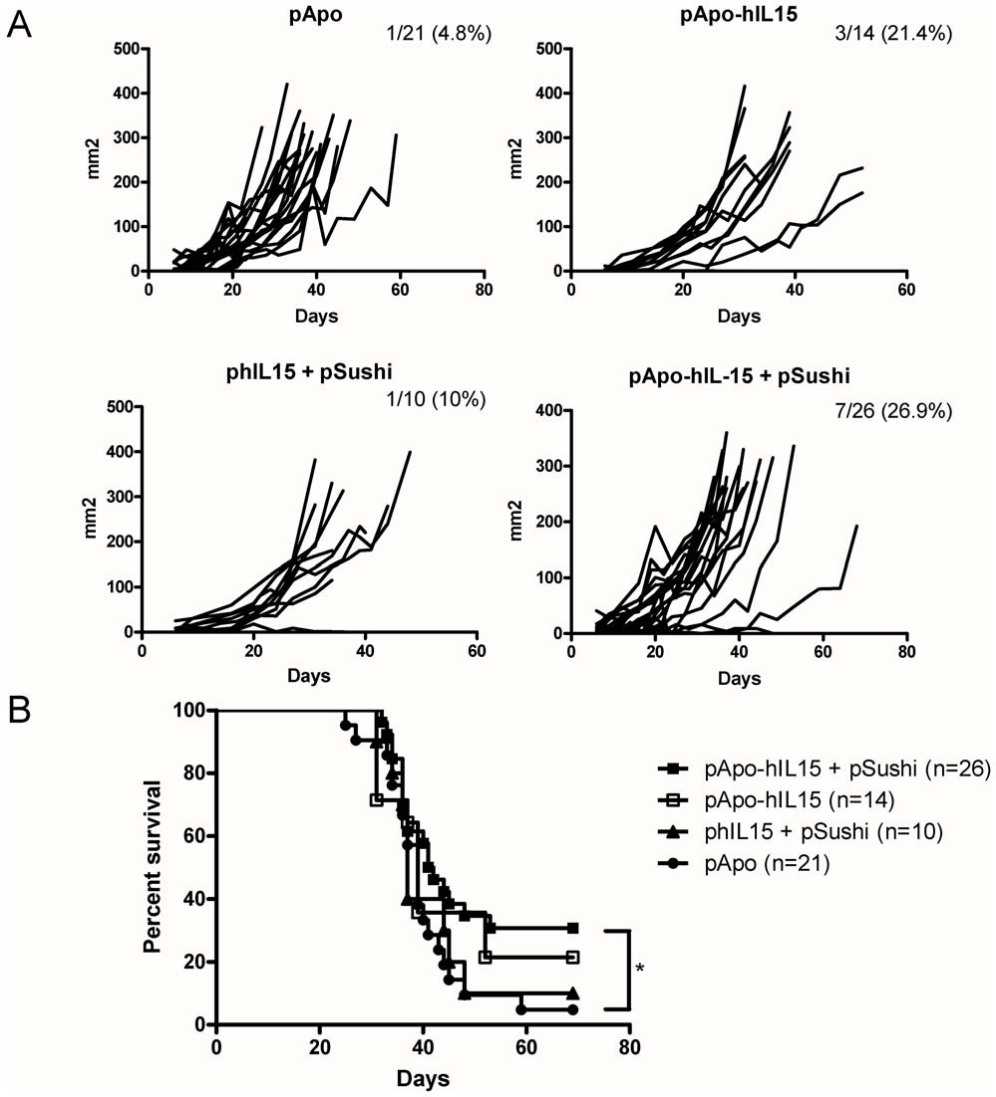
Immunotherapeutic activity of liver gene transfer with the APO-IL-15 constructions for established subcutaneous MC38 tumors. MC38 bearing mice were treated on day six after tumor cell inoculation with 10 µg of the indicated plasmids by hydrodynamic injection. (A) Individual follow-up of tumor sizes upon treatment with each plasmid. Graphics represent pooled data from 3 independent experiments. Fraction of surviving mice is indicated for each experimental group. Differences in tumor growth between the pApo-hIL15+ pSushi and the pApo group were found (p<0.001) in a nonlinear mixed effect model calculated with Monolix software. (B) Survival curves of the experimental groups from panel A (* indicates p<0.05 between pApo-hIL15+ pSushi and pApo in a Gehan-Breslow-Wilcoxon Survival Test).

Based on the observation of biological activity, we performed a series of experiments in mice grafted for six days with subcutaneous MC38-derived colon carcinomas. As can be seen in figure 6, a combination of pApo-hIL15+ pSushi resulted in 7 out of 26 complete rejections with an overall delay in tumor growth when compared to control groups. pApo-hIL15 cured 3 out of 14 animals while phIL15+ pSushi only 1 in 10. In the case of pApo A-I, one mouse rejected a tumor that could be related to the recently described non immune anti-tumor actions of Apo A-I [43]. Therapeutic effects resulted in extended survival as shown in figure 6B.

**Figure 7:**
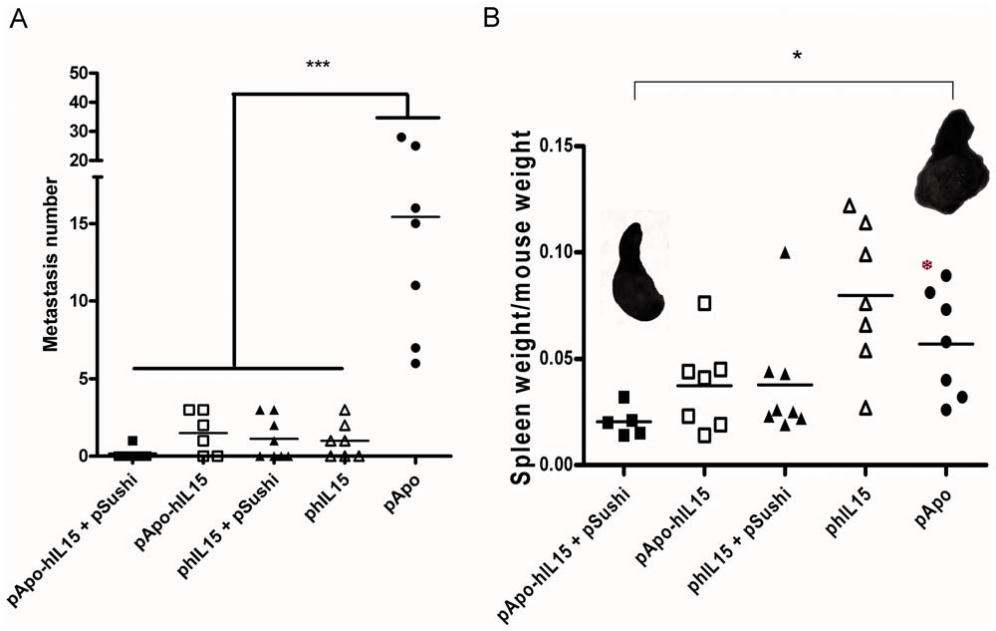
Antitumor effects following the transfer of IL-15 constructions against MC38 tumors metastatic to the liver. Mice injected intraesplenically with 5x10^5^ MC38 cells upon laparotomy were treated by hydrodynamic injections of the indicated plasmids one day later. On day 19 mice were euthanized and (A) liver surface was inspected to count macroscopic metastases observed in the surface of the liver under magnifying lens and data are represented on individual basis (*** indicates p<0.0001 between pApo-hIL15+ pSushi and the rest of the groups in Bonferroni test following one-way ANOVA test). (B) The weights of the spleens that also grafted tumors were recorded. Representative images of spleen tumors in the indicated groups are provided. The experiment shown is representative of two similarly performed (* indicates p<0.05 between pApo-hIL15+ pSushi and pApo groups in a t-student test).

Since our gene transfers are phenomena confined to some extent to the liver, it was conceivable that if MC38 were injected into the spleen to metastize to the liver via the portal vein, a more prominent therapeutic effect could then appear. Indeed, if mice that had been gene transferred on day 1 were euthanized on day 19 after tumor cell inoculation, the number of observable liver metastases was drastically reduced by all the plasmids encoding IL-15, but not by the pApo A-I expression plasmid. In the case of pApo-hIL15+ pSushi, 5 out of 6 mice were free of metastatic lesions on the liver surface (figure 7). Moreover, observations of the spleen tumors (measured as spleen weight/mouse weight) indicated that pApo-hIL15+ pSushi also markedly retarded the tumor at the primary site of malignant cell inoculation (figure 7B with inset photographs of representative spleen tumors). The pApo-hIL15 (without pSushi) and phIL15+ pSushi (without fusion to Apo A-I) showed a more partial effect on the splenic tumors as well. The role of the cellular immune system in these antitumor effects was confirmed because selective depletions with antibodies against CD8_β_, CD4 or NK cells hampered the therapeutic effects on the liver metastases (data not shown).

As a whole, these APO-IL-15 chimeric constructions transiently expressed by the liver give rise to therapeutic effects, which become candidates for improvement by means of combinatorial strategies [44].

## Discussion

This study intended to generate more stable and bioactive forms of IL-15 by incorporation into lipoproteins. Hydrodynamic injections offer a versatile technical opportunity to test these new agents without the need for cumbersome protein bio-production and purification procedures [45], [46]. In our case, the advantage was obvious because we are able to achieve the synthesis of our protein in the actual organ which produces HDL. Even though liver gene transfer faces obstacles for clinical translation, it is certainly a clinical alternative to be considered since it might be feasible and cost/effective [18], [46].

The idea of using Apo A-I fusions was originally used by our group to stabilize interferon α [33]. In the case of IL-15, this idea is likely to be more suitable since IL-15 is trans-presented [12], [22]. In essence, trans-presentation can be conceived to involve cell surface lipid bilayers and IL-15Rα [11], [42], [47]. Both conditions could be mimicked to some extent by the surface of an HDL lipoparticle, if IL-15Rα is complexed there to IL-15. Therefore, IL-15 could not only be stabilized but also become more bioactive, taking advantage in this way of its physiological mechanisms of action that depend on trans-presentation. It is of note that APO-IL-15 complexes only in part to HDL, so the free protein and the particle absorbed forms coexist in circulation, and maybe in equilibrium, although once complexed APO-IL-15 tends to remain attached to HDL at body temperature.

A series of studies had reported that the sushi domain region of IL-15Rα was required for trans-presentation and that the isolated sushi domain performs even more actively than the whole IL-15Rα protein for this cooperative function [13], [14], [15], hence giving a reason for our choice of this protein domain. Our results indicate that the complex pApo-hIL15:pSushi domain is stable and bioactive on NK and T cells *in vivo*.

The effects of gene transfer were able to correct in part the immune phenotype of IL-15Rα^−/−^ mice which have reduced CD8^+^ memory-phenotype T lymphocytes and virtually no NK or NKT cells. These findings underscore the biological potency of the constructions.

Our strategy has readily observed biological effects. However, direct side by side comparisons to other complexes of IL-15 such as the IL-15Rα-Fc conjugates [19], [21] or other previously reported constructs linking IL-15 to IL-15Rα [42] need to be carried out both in terms of pharmacokinetics and pharmacodynamics experiments. Indeed, our data with pAlbumin-hIL15 suggest that this fusion partner stabilizes IL-15 pharmacokinetics to a comparable extent, albeit the biodistribution and biological propierties may be quiet different.

Future research is required to optimize liver gene transfer and comparisons with the corresponding recombinant proteins are a must to ascertain the translational potential of these strategies. Using gene therapy and the liver to create a factory of proteins that complex into HDL has the advantage of using the natural routes of biosynthesis. However, the alternative of exogenously provided recombinant proteins is deemed more feasible for translation. In any case hydrodynamic gene transfers, although so far not translatable to humans, have provided important information on the pharmacokinetics and bioactivity of these new constructions. Indeed, although recombinant fusion proteins are our preferred approach to be translated into the clinic, we are further exploring the potential of liver gene transfer with gutless adenoviral vectors.

Interestingly, Apo A-I fusion cytokines incorporated to HDL do not impair the anti-inflammatory an cholesterol transporting functions of the HDL fractions, at least at the relative quantities generated in our studies. This has important implications for the overall strategy since as a result these agents are unlikely to result in increased cardiovascular risk.

IL-15 is being tested in patients as a monotherapy for melanoma and renal cell carcinoma in a dose escalating clinical trial (NCT01021059). However the use of IL-15 is more attractive when combined to adoptive T cell therapy [19], vaccines [20], [21] or immunostimulatory mAb [48]. We are currently testing various combinatorial approaches that include our APO-IL-15 constructs.

In a sense, the antitumor effects observed under our conditions of treatment are less efficacious than what we had predicted based on the intense proliferative effects on NK and T cells. However, MC38 is a difficult-to-treat tumor model [49] and therefore, reaching more than 25% curative efficacy in monotherapy against 6-day established MC38 tumors is a remarkable achievement, which would be, for instance, comparable to what agonist anti-CD137 mAb attain in the same context [50]. The experiments in mice bearing liver MC38 tumors are illustrative of the local potency of the approach against metastatic disease and are conceivably associated to the fact of *in situ* activation of intrahepatic lymphocytes. However we have observed that these numeric increases of lymphocytes at such organ do not correlate with brighter expression of effector or activation-denoting molecules (data not shown). Therefore, the provision in combinatorial immunotherapies of therapeutic tools to enhance tumoricidal activity on cell per cell basis is guiding our next steps (MC Ochoa *et al.* unpublished results). In any case, it is of note that unresectable liver metastases of colon cancer constitute a major unmet therapeutic need and that our results show evidence for therapeutic efficacy in difficult to treat mouse models.

All in all, we have constructed chimeric IL-15 variants that are incorporated at least in part into HDL. In our experiments, we took advantage of IL-15 trans-presentation by the sushi domain of IL-15Rα to attain stronger bioactivity. Partial antitumor efficacy as a monotherapy strongly advocates for further development with combinatorial approaches, both in terms of adding other immunotherapeutic agents and in terms of further engineering the cytokine transgene products to be built into HDL.

## Supporting Information

Figure S1 **Schematic representation of the plasmids used for hydrodynamic injections.** pcDNA3.1-based expression plasmids were engineered to encode for cassettes of expression encompassing Apo A-I lipoprotein, Apo A-I fused to human IL-15 and the sushi domain of IL-15Rα. Human IL-15 was cloned in a pIRES1neo- based plasmid
(TIF)

Figure S2 **Expression of Sushi mRNA in the liver of mice receiving hydrodynamic injections with pApo-hIL15 and the plamid encoding the IL-15Rα sushi domain.** Mice received 10 µg of pApo-hIL15 or pApo-hIL15+ pSushi by hydrodynamic injection and 12 h following treatment, livers were harvested and expression of Sushi mRNA was studied by quantitative RT-PCR normalized to β-actin. Data from 2 mice per group are presented.
(TIF)

Figure S3 **Similar kinetics of hIL-15 serum concentrations in mice hydrodynamically gene-transferred with pAlbumin-hIL15 and pApo-hIL15.** C56Bl/6 mice received a hydrodynamic injection with 10 µg of a construction coding for a protein encompassing albumin and hIL-15 or pApo-hIL15. Sera were obtained at the indicated times and hIL-15 concentrations were measured by ELISA.
(TIF)

Figure S4 **HDL-fractions from pApo-hIL15 injected mice preserve their normal physiological functions.** (A) The endothelial Py4-1 cell line was cultured in presence or absence of TNF-α for 4 h and later incubated or not overnight with HDL from mice injected with pApo or pApo-hIL15. Expression of VCAM was examined by flow cytometry. (B) 3T3 L1 cells were loaded by incubation with 3H-cholesterol and then HDLs from mice treated with pApo, pApo-hIL15 or pApo-hIL15+ pSushi were added to the culture. Cholesterol efflux was measured as 3H cpms in the culture supernatans.
(TIF)

Figure S5 **Percentage of CD8 and NK cells in spleen, liver and blood increases in response to liver gene co-transfer of Apo-IL-15 and IL-15Rα sushi domain.** Time course analyses of cell suspensions from spleen (A), liver (B) and blood (C) defining the percentages of lymphocyte subsets at the indicated time points (* indicates p<0.05 and **p<0.01 between pApo-hIL15+ pSushi and phIL15+ pSushi group in a Bonferroni test following two-way ANOVA test with p<0.0001). This experiment is representative of two performed with 2–3 mice for each time point. In (B) mice were sequentially followed.
(TIF)

Figure S6 **Apo-IL-15 and IL-15Rα sushi domain gene transfer to the liver increases NK activity in the spleen.** Natural cytotoxicity of spleen cells against the classical NK target Yac-1 at the indicated E:T ratios. Effector cells were obtained from mice treated 3 days earlier with the indicated plasmids or plamid combination. Data are from one representative experiment out of two performed. Each experiment was performed with two mice per group and rendered similar results.
(TIF)

Figure S7 **Apo-IL-15 and IL-15Rα sushi domain gene transfer to the liver induces the proliferation CD8 and NK lymphocytes measured by BrdU incorporation.** Mice were gene-transferred by hydrodynamic injections of the indicated plasmids. Subsequently three daily ip doses of BrdU were provided. Incorporation of BrdU to DNA was measured at 72 h since the gene transference by immunostaining and flow-cytometry.Gating strategy (A and C) and data of an experiment with two mice per group (B and D) are showed.
(TIF)

Figure S8 **NK and CD8 memory-phenotype T cells augment in IL15Rα−/− mice on a B6129S background upon combined liver gene transfer of pApo-IL-15 and pSushi.** (A and B) B6129S WT and syngenic IL15Rα−/− mice were treated by hydrodynamic injections of the indicated plasmids 3 times every 7 days. At day 25 mononuclear leukocytes from the spleen were immunostained. Dots represent individual data from a single experiment with five mice per group. (C) Represents a separate experiment as in A/B that compares pSushi with a full length IL15Rα version upon coinjection with pApo-hIL15 to rescue the NK and CD8 phenotype of IL-15Rα−/− mice.
(TIF)
