# Supplementary figures and images for: Liver Gene Transfer of Interkeukin-15 Constructs That Become Part of Circulating High Density Lipoproteins for Immunotherapy
Source: PLoS One. 2012 Dec 21;7(12):e52370. doi: 10.1371/journal.pone.0052370 (PMC3528770; doi:10.1371/journal.pone.0052370)

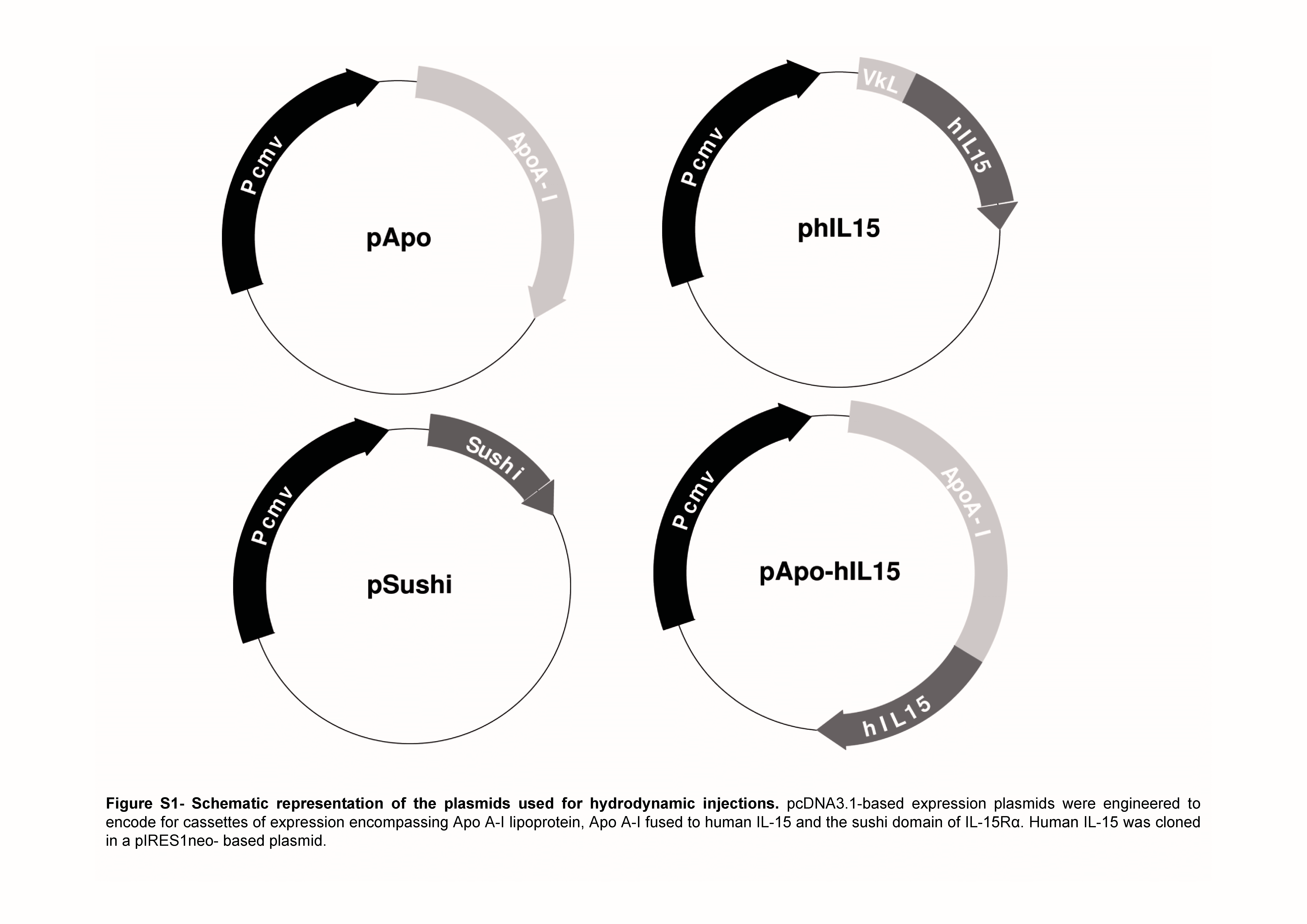

Supplement: Figure S1 — Schematic representation of the plasmids used for hydrodynamic injections. pcDNA3.1-based expression plasmids were engineered to encode for cassettes of expression encompassing Apo A-I lipoprotein, Apo A-I fused to human IL-15 and the sushi domain of IL-15Rα. Human IL-15 was cloned in a pIRES1neo- based plasmid (TIF) [file pone.0052370.s001.tif]

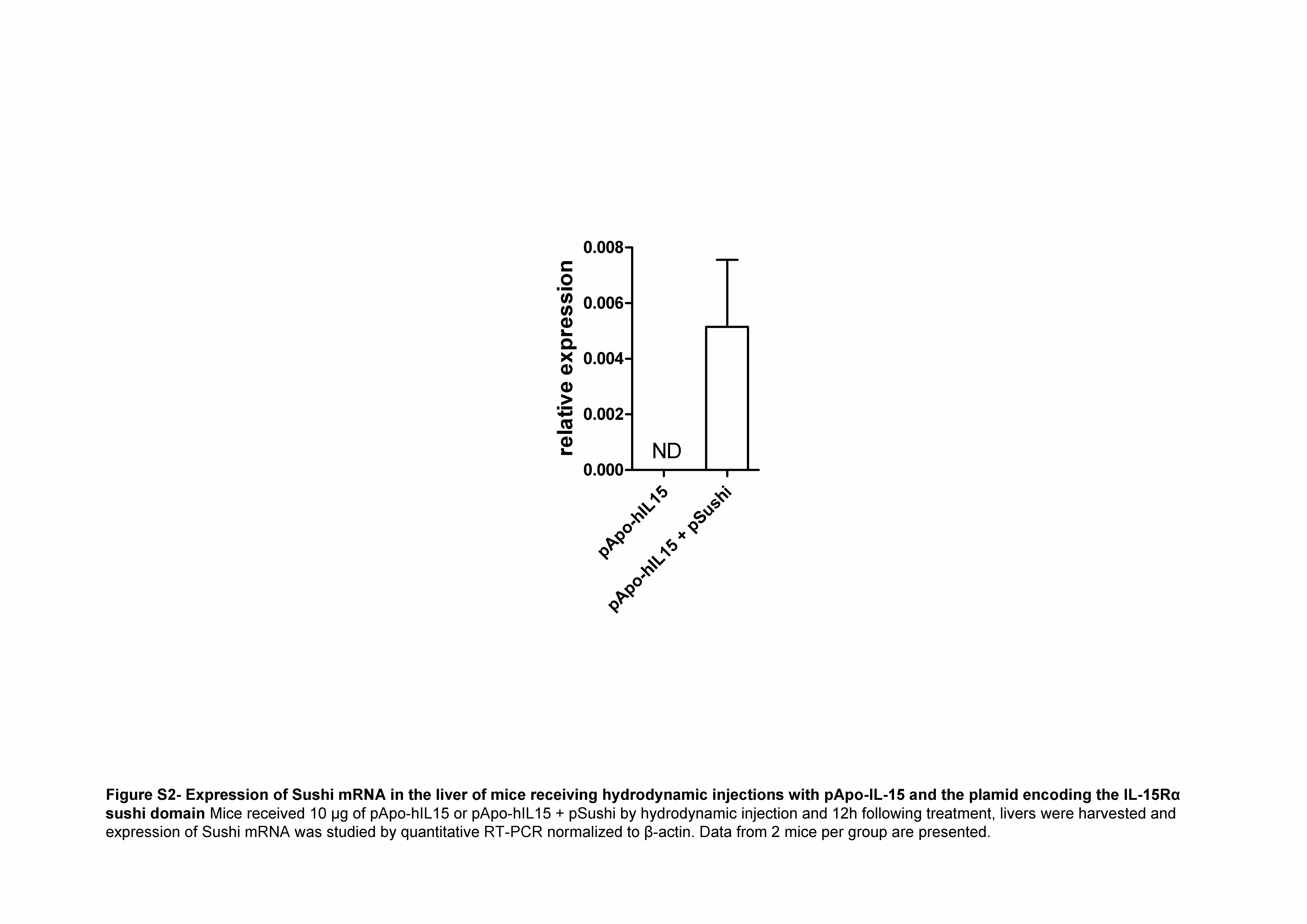

Supplement: Figure S2 — Expression of Sushi mRNA in the liver of mice receiving hydrodynamic injections with pApo-hIL15 and the plamid encoding the IL-15Rα sushi domain. Mice received 10 µg of pApo-hIL15 or pApo-hIL15+ pSushi by hydrodynamic injection and 12 h following treatment, livers were harvested and expression of Sushi mRNA was studied by quantitative RT-PCR normalized to β-actin. Data from 2 mice per group are presented. (TIF) [file pone.0052370.s002.tif]

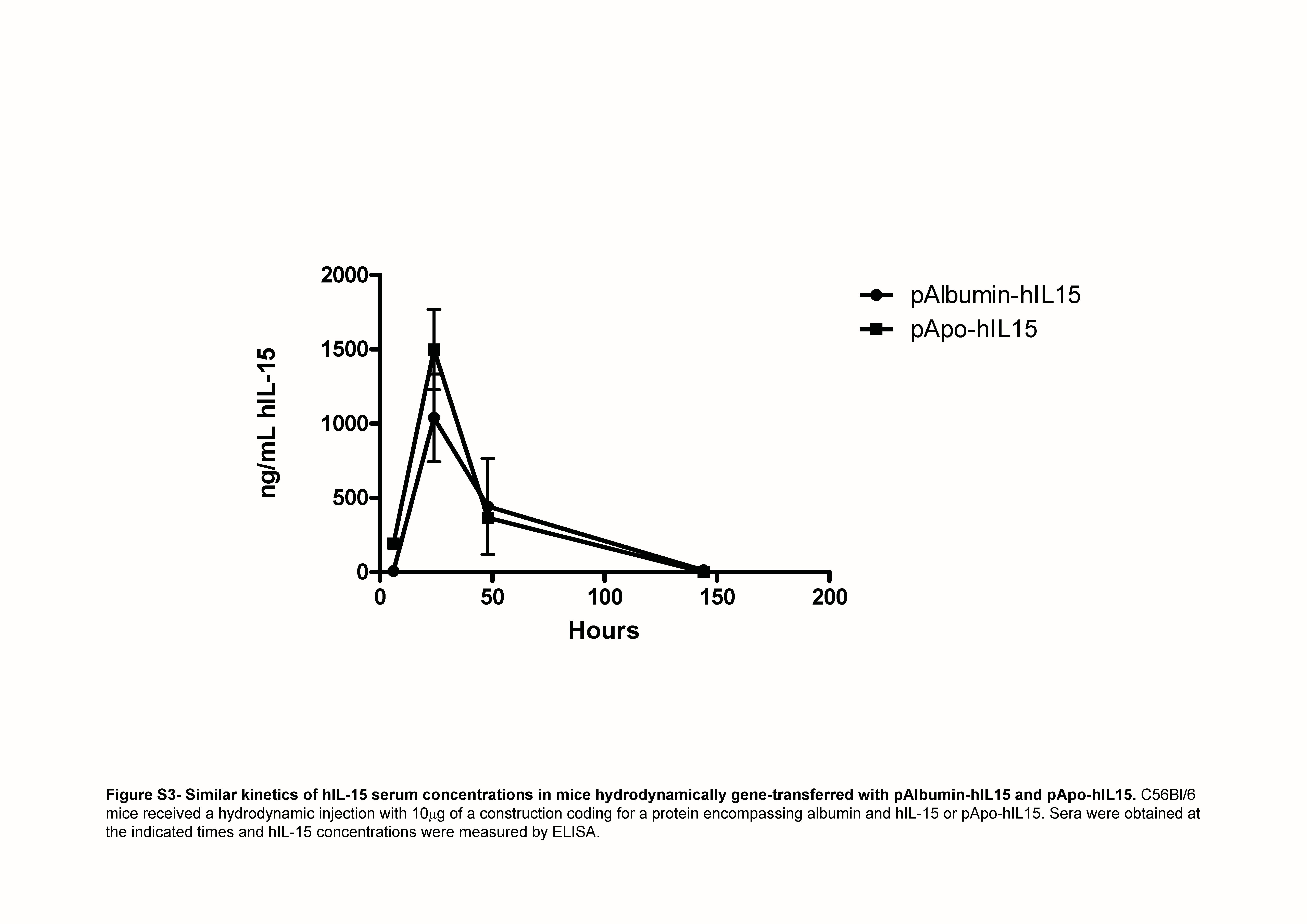

Supplement: Figure S3 — Similar kinetics of hIL-15 serum concentrations in mice hydrodynamically gene-transferred with pAlbumin-hIL15 and pApo-hIL15. C56Bl/6 mice received a hydrodynamic injection with 10 µg of a construction coding for a protein encompassing albumin and hIL-15 or pApo-hIL15. Sera were obtained at the indicated times and hIL-15 concentrations were measured by ELISA. (TIF) [file pone.0052370.s003.tif]

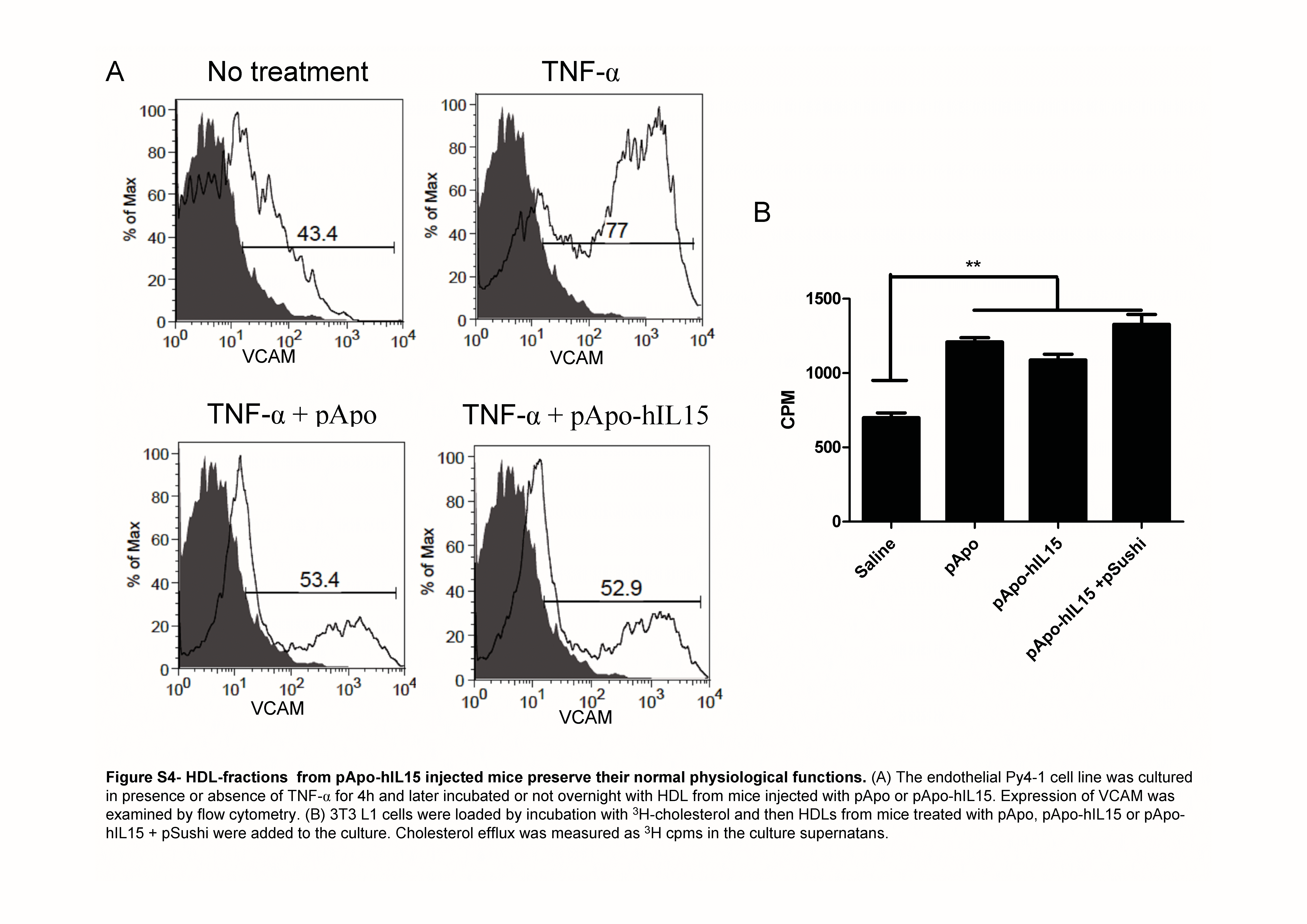

Supplement: Figure S4 — HDL-fractions from pApo-hIL15 injected mice preserve their normal physiological functions. (A) The endothelial Py4-1 cell line was cultured in presence or absence of TNF-α for 4 h and later incubated or not overnight with HDL from mice injected with pApo or pApo-hIL15. Expression of VCAM was examined by flow cytometry. (B) 3T3 L1 cells were loaded by incubation with 3H-cholesterol and then HDLs from mice treated with pApo, pApo-hIL15 or pApo-hIL15+ pSushi were added to the culture. Cholesterol efflux was measured as 3H cpms in the culture supernatans. (TIF) [file pone.0052370.s004.tif]

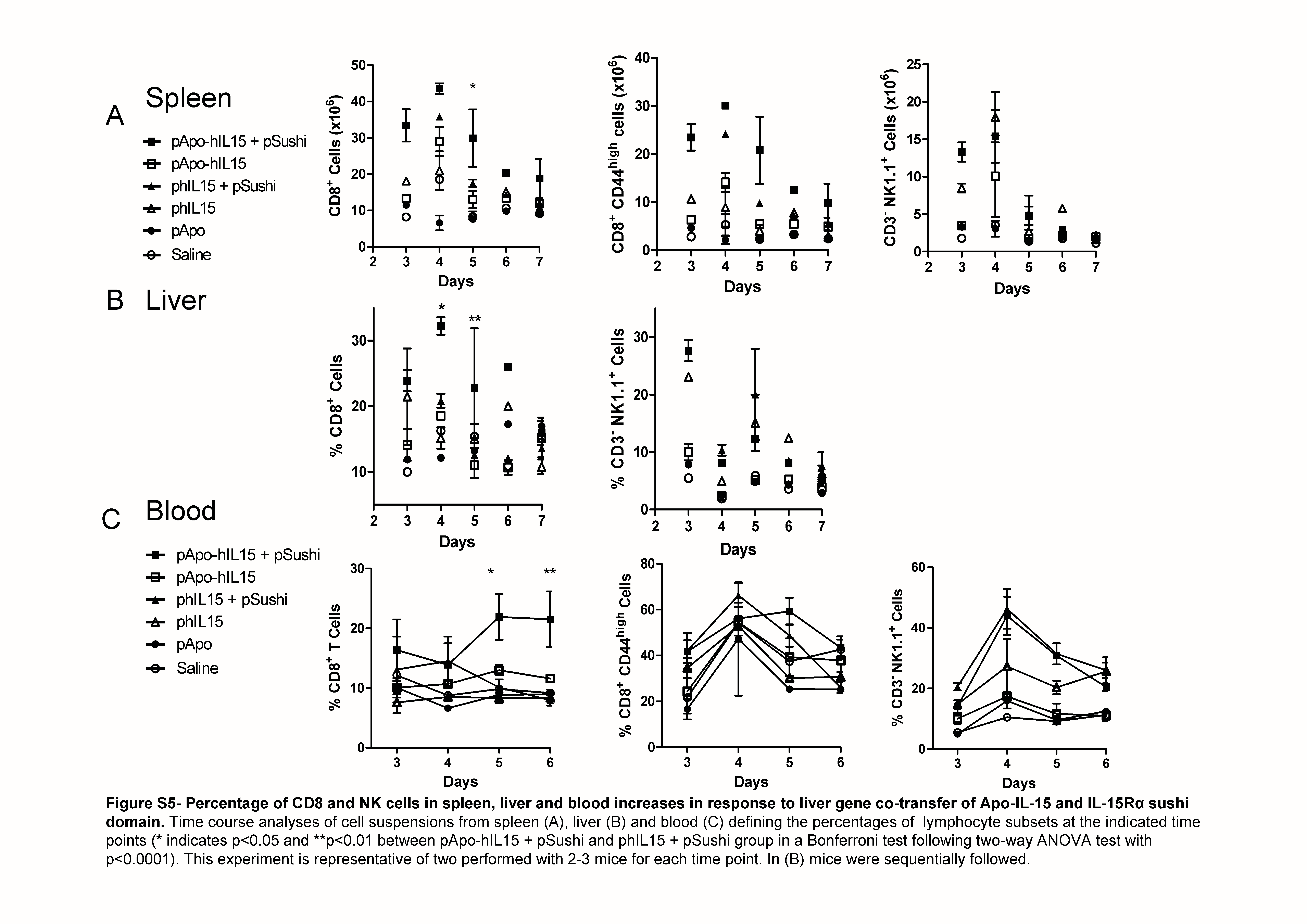

Supplement: Figure S5 — Percentage of CD8 and NK cells in spleen, liver and blood increases in response to liver gene co-transfer of Apo-IL-15 and IL-15Rα sushi domain. Time course analyses of cell suspensions from spleen (A), liver (B) and blood (C) defining the percentages of lymphocyte subsets at the indicated time points (* indicates p<0.05 and **p<0.01 between pApo-hIL15+ pSushi and phIL15+ pSushi group in a Bonferroni test following two-way ANOVA test with p<0.0001). This experiment is representative of two performed with 2–3 mice for each time point. In (B) mice were sequentially followed. (TIF) [file pone.0052370.s005.tif]

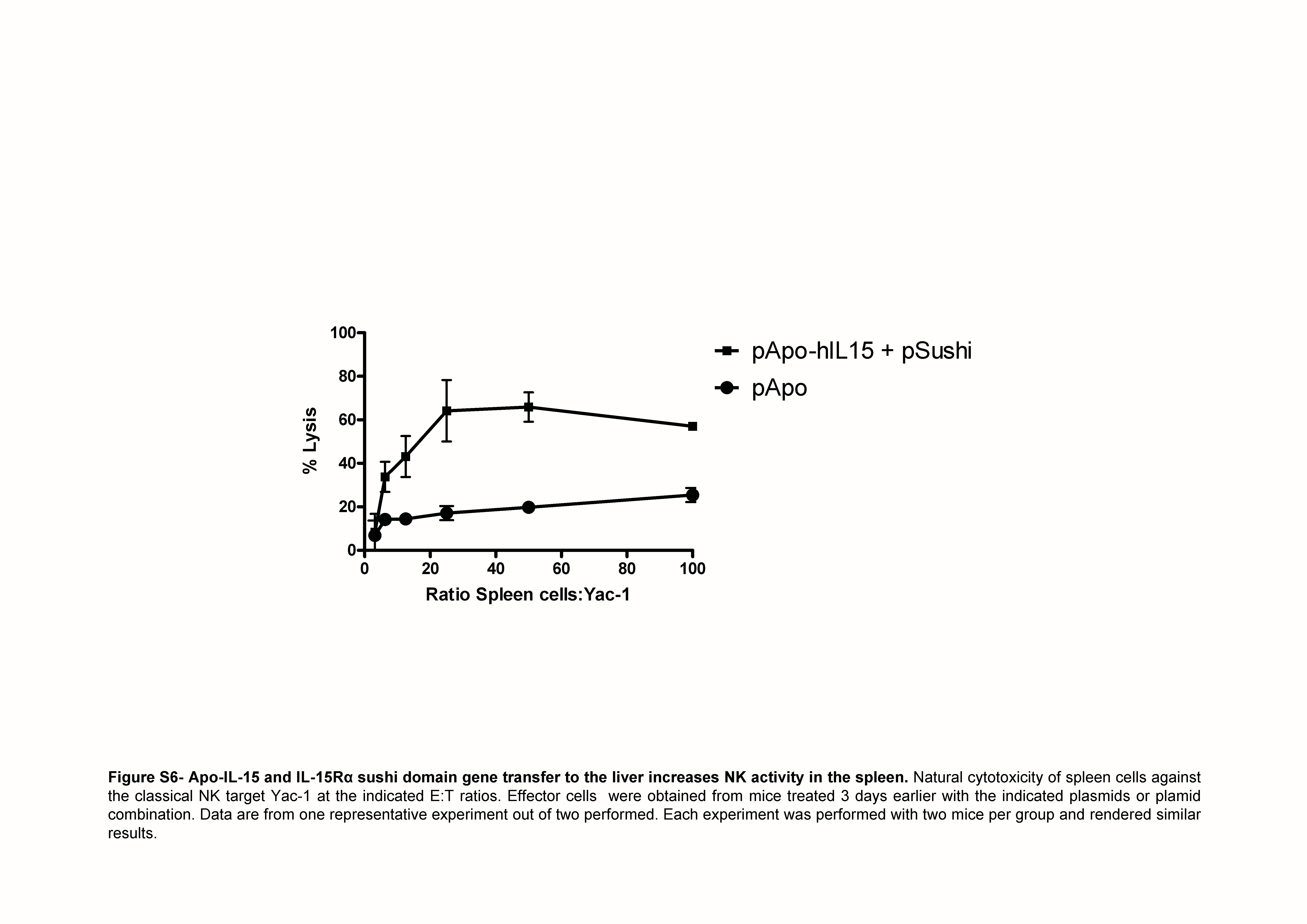

Supplement: Figure S6 — Apo-IL-15 and IL-15Rα sushi domain gene transfer to the liver increases NK activity in the spleen. Natural cytotoxicity of spleen cells against the classical NK target Yac-1 at the indicated E:T ratios. Effector cells were obtained from mice treated 3 days earlier with the indicated plasmids or plamid combination. Data are from one representative experiment out of two performed. Each experiment was performed with two mice per group and rendered similar results. (TIF) [file pone.0052370.s006.tif]

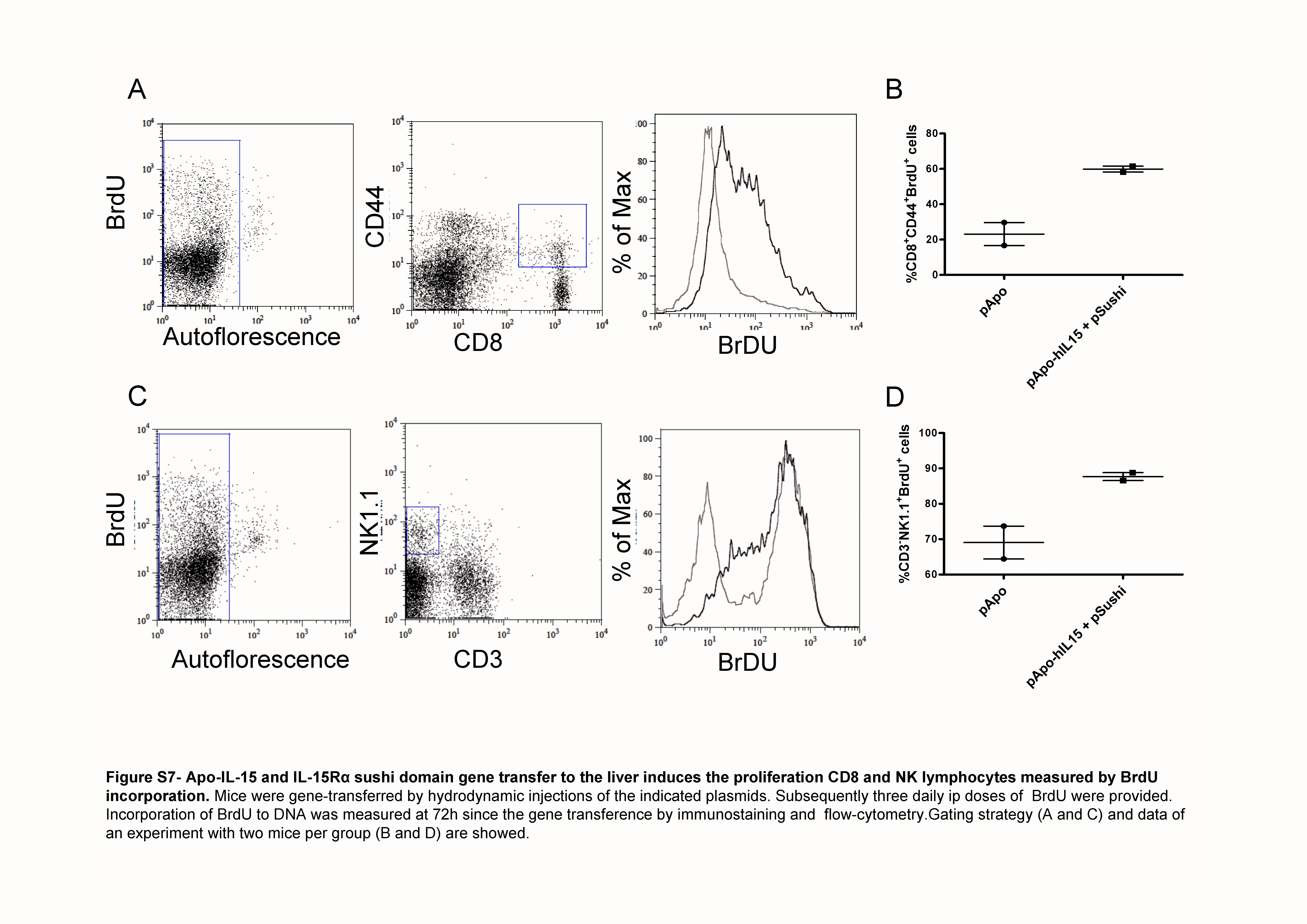

Supplement: Figure S7 — Apo-IL-15 and IL-15Rα sushi domain gene transfer to the liver induces the proliferation CD8 and NK lymphocytes measured by BrdU incorporation. Mice were gene-transferred by hydrodynamic injections of the indicated plasmids. Subsequently three daily ip doses of BrdU were provided. Incorporation of BrdU to DNA was measured at 72 h since the gene transference by immunostaining and flow-cytometry.Gating strategy (A and C) and data of an experiment with two mice per group (B and D) are showed. (TIF) [file pone.0052370.s007.tif]

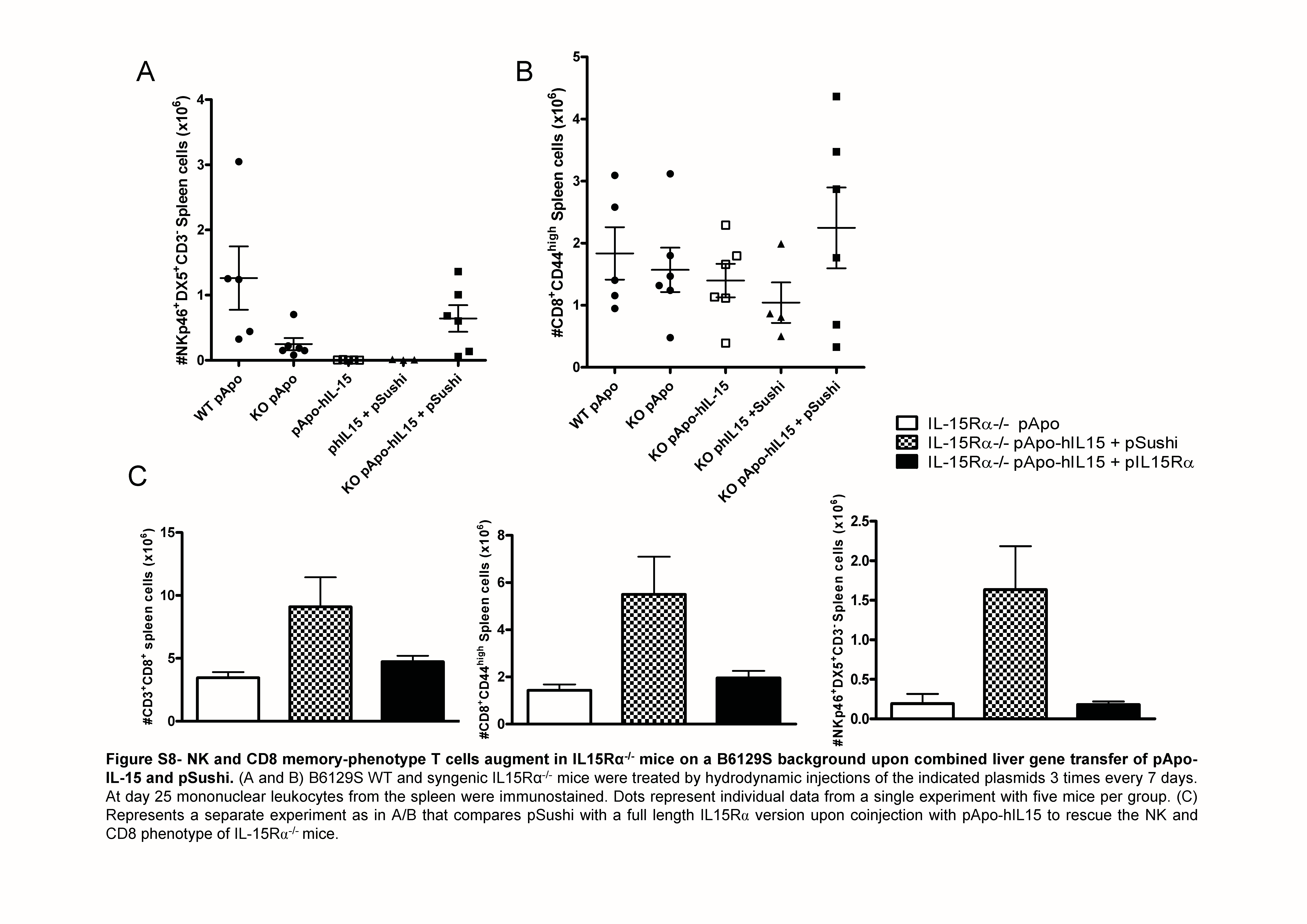

Supplement: Figure S8 — NK and CD8 memory-phenotype T cells augment in IL15Rα−/− mice on a B6129S background upon combined liver gene transfer of pApo-IL-15 and pSushi. (A and B) B6129S WT and syngenic IL15Rα−/− mice were treated by hydrodynamic injections of the indicated plasmids 3 times every 7 days. At day 25 mononuclear leukocytes from the spleen were immunostained. Dots represent individual data from a single experiment with five mice per group. (C) Represents a separate experiment as in A/B that compares pSushi with a full length IL15Rα version upon coinjection with pApo-hIL15 to rescue the NK and CD8 phenotype of IL-15Rα−/− mice. (TIF) [file pone.0052370.s008.tif]
